# *Lacticaseibacillus paracasei* DG enhances the lactoferrin anti-SARS-CoV-2 response in Caco-2 cells

**DOI:** 10.1080/19490976.2021.1961970

**Published:** 2021-08-07

**Authors:** Claudio Salaris, Melania Scarpa, Marina Elli, Alice Bertolini, Simone Guglielmetti, Fabrizio Pregliasco, Paola Brun, Ignazio Castagliuolo

**Affiliations:** a Department of Molecular Medicine, University of Padua, Padua, Italy; b Laboratory of Advanced Translational Research, Veneto Institute of Oncology IOV - IRCCS, Padua, Italy; c AAT-Advanced Analytical Technologies S.r.l., Fiorenzuola d’Arda, Piacenza, Italy; d Department of Food, Environmental and Nutritional Sciences (Defens), University of Milan, Milan, Italy; e IRCCS Istituto Ortopedico Galeazzi, University of Milan, Milan, Italy

**Keywords:** Probiotics, lactobacilli, lactoferrin, sars-Cov-2, antiviral immunity, covid-19

## Abstract

The novel severe acute respiratory syndrome coronavirus 2 (SARS-CoV-2) is causing the ongoing global pandemic of coronavirus disease 2019 (COVID-19), which primarily manifests with respiratory distress and may also lead to symptoms associated with the gastrointestinal tract. Probiotics are living microorganisms that have been shown to confer immune benefits. In this study, we investigated the immunomodulatory effects and anti-SARS-CoV-2 activity of three different *Lacticaseibacillus* probiotic strains, either alone or in combination with lactoferrin, using the intestinal epithelial Caco-2 cell line. Our results revealed that the *Lacticaseibacillus paracasei* DG strain significantly induced the expression of genes involved in protective antiviral immunity and prevented the expression of proinflammatory genes triggered by SARS-CoV-2 infection. Moreover, *L. paracasei* DG significantly inhibited SARS-CoV-2 infection *in vitro. L. paracasei* DG also positively affected the antiviral immune activity of lactoferrin and significantly augmented its anti-SARS-CoV-2 activity in Caco-2 intestinal epithelial cells. Overall, our work shows that the probiotic strain *L. paracasei* DG is a promising candidate that exhibits prophylactic potential against SARS-CoV-2 infection.

## Introduction

Severe acute respiratory syndrome coronavirus 2 (SARS-CoV-2), an enveloped virus with a single-stranded positive-sense RNA genome,^1^ is a novel coronavirus that generated a pandemic outbreak, designated coronavirus disease 2019 (COVID-19), which was initially identified in Wuhan, China, in December 2019 and spread rapidly worldwide.^2^ COVID-19 has emerged as a multifaceted, multisystem and multiorgan disorder ranging from nonspecific flu-like symptoms to pneumonia, acute respiratory distress syndrome (ARDS), multiple organ failure and death.^2,3^ SARS-CoV-2 infection starts by the binding of the virus spike surface glycoprotein (SgP) to host cell surface heparan sulfate proteoglycans (HSPGs) and angiotensin-converting enzyme 2 (ACE2) receptors present on many human cells, which are then cleaved by host proteases, thus allowing virus internalization into host cells.^4^ SARS-CoV-2 appears to primarily spread through respiratory droplets and secretions, but the gastrointestinal tract could be another potential route of infection, since in approximately 17% of cases, gastrointestinal disorders are associated with respiratory symptoms.^5^ These data suggest that the gastrointestinal tract might be a location of viral activity and replication, in line with the high expression of ACE2 in the intestinal epithelium.^6^

Currently, therapeutic interventions against SARS-CoV-2 infection rely on supportive care and symptom alleviation, with just one conditionally authorized antiviral treatment regimen for COVID-19^7^ and several effective specific treatments still under investigation.^8–10^ Emergency use of four vaccines has been recently authorized^7,^ while several other vaccine candidates are still being evaluated in phase III trials.^11^ Thus, novel preventative and treatment strategies for SARS-CoV-2 infection are crucial to relieve the public health, economic and societal impacts of the virus.

Probiotics, defined as live microorganisms that when administered in adequate amounts, confer a health benefit on the host,^12^ act on both the innate and acquired immune systems and have the potential to decrease the severity of infections in the gastrointestinal^13^ and upper respiratory tracts.^14^ They exert antiviral activity by direct probiotic–virus interactions, production of antiviral inhibitory metabolites, and stimulation of the type I interferon response and antibody production against viruses.^15–17^ The potential for probiotics to reduce the risk and severity of viral respiratory tract infections is also supported by clinical and experimental studies on influenza, rhinovirus, and respiratory syncytial virus.^18,19^ Although none of these effects have been tested with SARS-CoV-2, some probiotic strains do have antiviral activity against other coronaviruses.^20–22^ The Lactobacillaceae family includes most of the microbial strains used in probiotic food and supplements.^23^ It consists of gram-positive fermentative bacteria, including species that are important members of the microbial ecosystems of human mucosae, such as those of the intestinal tract and vagina.^24^ Considering the important contribution that these microorganisms may exert for host immunity, it has been suggested to provide COVID-19 patients with nutritional support and prebiotic or probiotic supplementation to re-normalize the intestinal microbiota community structure and decrease the risk of infection.^25^ However, no study has addressed whether *Lactobacillus* spp. can affect intestinal antiviral immunity and SARS-CoV-2 infection.

Lactoferrin (LF) is a naturally occurring multifunctional glycoprotein with broad-spectrum antiviral, immunomodulatory and anti-inflammatory effects.^26,27^ LF was shown to block multiple common human coronaviruses and SARS-CoV-2 *in vitro*^28,29^ by preventing the interaction between the viral particle and its cell receptors, represented by HSPGs,^30^ which act as necessary cofactors for SARS-CoV-2 infection.^31^

The studies supporting the immune benefits and antiviral activity of probiotics prompted us to hypothesize that *Lacticaseibacillus* strains could affect the antiviral immune response and SARS-CoV-2 infection of intestinal cells. Therefore, the present work aimed to investigate the *in vitro* antiviral immunomodulatory effects of three different *Lacticaseibacillus* strains, either alone or in combination with LF. Furthermore, their antiviral activity was evaluated in an *in vitro* model of SARS-CoV-2 infection. Based on our findings, we propose that the *Lacticaseibacillus paracasei* DG strain is a promising probiotic useful for the prevention of SARS-CoV-2 infection and alleviation of associated symptoms.

## Materials and methods

### Cells, viruses, bacterial strains and reagents

The human colon adenocarcinoma cell line Caco-2 (ATCC®HTB-37™) and monkey kidney epithelial cell line Vero E6 (ATCC®CRL-1586™) were grown in DMEM (Gibco-Thermo Fisher Scientific, Waltham, USA) supplemented with 10% (v/v) fetal bovine serum (FBS), 1% (v/v) sodium pyruvate and 1% (v/v) penicillin/streptomycin (all from Gibco-Thermo Fisher Scientific, Waltham, USA) at 37°C in a humidified incubator containing 5% CO_2_.

Lactobacillus strains, namely, *Lacticaseibacillus rhamnosus* (formerly *Lactobacillus rhamnosus*) GG (ATCC 53103), *L. paracasei* (formerly *Lactobacillus paracasei*) DG (CNCM I-1572; *L. paracasei* DG®; Enterolactis®, SOFAR S.p.A.) and *L. paracasei* LPC-S01 (DSM 26760) were cultured on De Man Rogosa Sharpe (MRS) plates (Difco, BD). The strains were incubated for 72 h at 37°C under anaerobic conditions. Strain ATCC 53103 was purchased from the American Type Culture Collection (ATCC), while strains DG and LPC-S01 were provided by Sofar S.p.A. (Milan, Italy). LF was acquired as Globoferrina® (Sofar, Italy). LF was used alone and/or in combination with the probiotics at a final concentration of 100 µg/ml. Sterile DMEM (Gibco-Thermo Fisher Scientific, Waltham, USA) supplemented with 20% glycerol was added as a control test. Remdesivir (Apollo Scientific, Bredbury, UK) was used as an antiviral control for our assays. Remdesivir was used alone for 3 h at a final concentration of 10 μM.

### Viral stock preparation and titration

SARS-CoV-2 was isolated from a patient at the Microbiology Unit, University Hospital Padua. The viral strain was propagated and titrated as previously described.^28^ See Supplementary Methods for details. All the infection experiments were performed in a biosafety level 3 (BSL-3) laboratory at the Department of Molecular Medicine, University of Padova, Padova, Italy.

### Preparation of bacterial strains

Broth cultures were prepared in MRS broth with incubation for 18 h at 37°C under anaerobic conditions. Following incubation, the strains were centrifuged for 10 min at 3000 rpm, and the cell pellets were washed twice with sterile distilled water. The optical density at 600 nm (OD_600_) of washed cultures was adjusted to 0.3 to reach 2.5 × 10^6^ colony-forming units (CFU) in a 20 µl volume. Standardized washed cultures were serially diluted for viable counts and centrifuged for ten minutes at 3000 rpm. The pellets were resuspended in sterile DMEM (Gibco-Thermo Fisher Scientific, Waltham, USA) supplemented with 20% glycerol (Merck).

### Caco-2 cell culture and treatments

Caco-2 cells were seeded in 12-well plates (2 × 10^5^ cells/mL). After reaching confluence, the cells were washed in phosphate-buffered saline (PBS; Gibco-Thermo Fisher Scientific, Waltham, USA) and incubated in antibiotic-free medium (AFM) or subjected to one of the following treatments described below, according to the experimental design displayed in Supplementary Figure S1. In all treatment protocols, confluent Caco-2 cells were supplemented with the bacterial strain (multiplicity of infection (MOI) of 10:1 bacteria: cells). LF was added at a concentration of 100 μg/ml with or without the bacterial strain. After 3 h, in the probiotic treatment experiments, cells were washed in PBS (Gibco-Thermo Fisher Scientific, Waltham, MA, USA) and incubated with fresh medium supplemented with antibiotics (penicillin/streptomycin). The cells were harvested 24 h later for RNA extraction. In the probiotic pre-infection treatment protocol, cells were washed in PBS (Gibco-Thermo Fisher Scientific, Waltham, MA, USA), supplemented with fresh medium with antibiotics (penicillin/streptomycin) and infected with SARS-CoV-2 (MOI 2:1) for 1 h. Twenty-four hours post infection, the cells were harvested for RNA extraction, and the supernatants were harvested for viral titration.

### RNA extraction and real-Time RT-PCR

Total RNA was isolated, and gene expression analysis was performed as previously reported.^28^ The specific forward and reverse primers used are summarized in Supplementary Table S1. Data are presented as the mean fold change over the control.

### Immunofluorescence

Caco-2 cells were seeded in sterile coverslips inside 6-well plates. After reaching confluence, the cells were subjected to one of the previously described treatments and infected with SARS-CoV-2 at an MOI of 2:1. Staining was performed as previously described.^28^ See Supplementary Methods for details.

### Statistical analysis

All experiments were performed in duplicate wells for each condition and repeated at least three times. Data are shown as the mean +/− SD. Statistical analysis was performed using GraphPad Prism Software 6.0 (GraphPad Software Inc., La Jolla, USA). Comparisons were performed using one-way ANOVA for multiple comparisons as indicated in each figure legend. Differences among and between individual groups were compared as indicated in each figure legend, with P ≤ 0.05 considered significant.

## Results

### Probiotic Lacticaseibacillus strains increase the antiviral immune response in vitro

**Figure 1.**
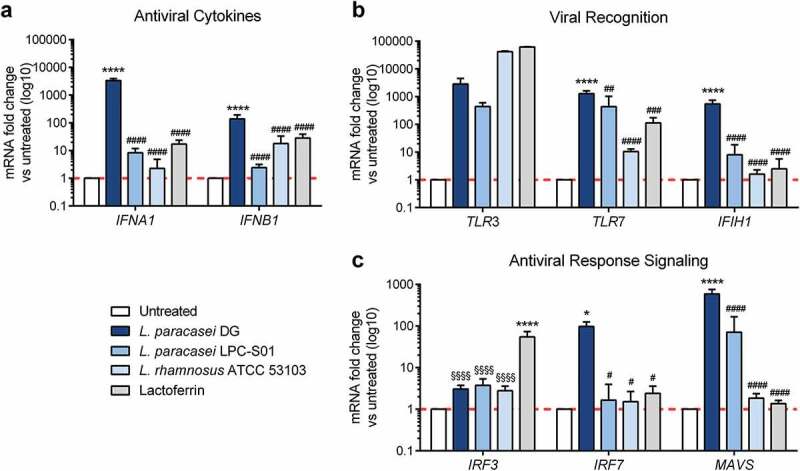
***Lacticaseibacillus* probiotic strains enhance the antiviral immune response *in vitro***. The gene expression of (a) type I interferons, (b) innate immune receptors and (c) regulatory molecules of the innate immune response was assessed by real-time qPCR in Caco-2 cells 24 h after a 3 h treatment with *Lacticaseibacillus* probiotic strains (n = 5). Data are shown as the relative fold change compared to the untreated control (arbitrarily set as 1) and presented as the mean ± SD. *P < .05 ****P < .0001 vs untreated; ^#^*P* < .05, ^##^*P* < .01, ^###^*P* < .001, ^####^*P* < .0001 vs *L. paracasei DG*; and ^§§§§^*P* < .0001 vs LF based on one-way ANOVA (followed by Bonferroni’s multiple comparison test)

The antiviral immunomodulatory effects of probiotic *Lacticaseibacillus* strains were evaluated *in vitro* using the human intestinal epithelial cell line Caco-2. LF, a common nutritional supplement known for its immunomodulatory properties, was also tested for reference. As shown in Figure 1, probiotic treatment induced significant changes in the expression profile of several genes involved in the antiviral immune response. The levels of the antiviral cytokines interferon alpha (*IFNA1*) and beta (*IFNB1*) were significantly enhanced by *L. paracasei* DG (Figure 1a). Moreover, *L. paracasei* DG significantly augmented the expression of *TLR7*, a pattern recognition receptor involved in RNA virus sensing; *IFIH1*, the gene encoding MDA5, which is a molecular sensor of viral RNA; and *IRF7* and *MAVS*, which participate in antiviral response signaling pathways (Figure 1b and 1c). Notably, *L. paracasei DG* significantly triggered the upregulation of *IFNA1, IFNB1, TLR7, IFIH1, IRF7* and *MAVS* transcript levels compared to those with the other probiotic strains, which showed a trend toward the upregulation of the aforementioned genes. These results reveal that all three probiotic strains can stimulate different levels of antiviral immunity activity, with *L. paracasei* DG being the most promising in terms of significant upregulation of the expression of all the genes tested.

### Antiviral immune response activity of probiotic Lacticaseibacillus strains and lactoferrin in combination in vitro

**Figure 2.**
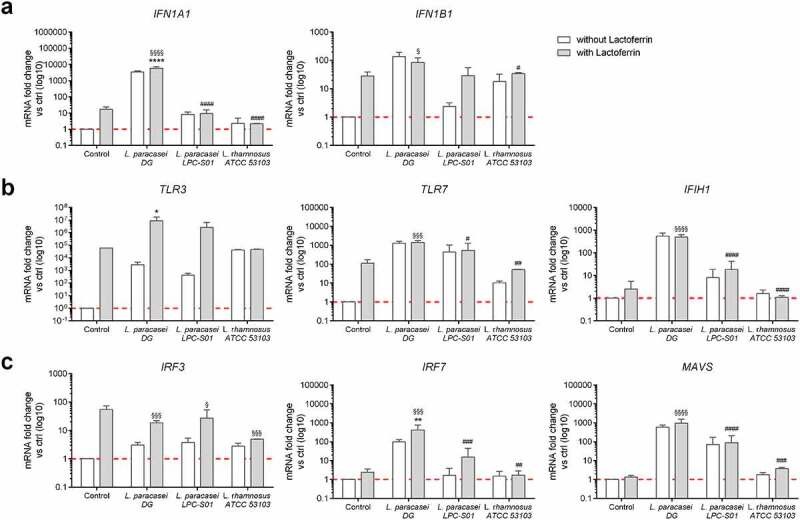
**Effect of the lactoferrin and probiotic combination on the antiviral immune response *in vitro***. The gene expression of (a) type I interferons, (b) innate immune receptors and (c) regulatory molecules of the innate immune response was assessed by real-time qPCR in Caco-2 cells 24 h after a 3 h treatment with or without lactoferrin in combination with *Lacticaseibacillus* probiotic strains (n = 5). Data are shown as the relative fold change compared to the untreated control (arbitrarily set as 1) and presented as the mean ± SD. *P < .05, **P < .01, ****P < .0001 vs *L. paracasei DG* without LF; ^#^*P* < .05, ^##^*P* < .01, ^###^*P* < .001, ^####^*P* < .0001 vs *L. paracasei DG* with LF; and ^§^*P* < .05, ^§§§^*P* < .001, ^§§§§^*P* < .0001 vs LF based on one-way ANOVA (followed by Bonferroni’s multiple comparison test)

Considering the ability of LF to stimulate the antiviral immune response,^28^ its effectiveness when used in combination with probiotic *Lacticaseibacillus* strains was evaluated. When Caco-2 cells were treated with LF and *L. paracasei* LPC S01 in combination or with LF and *L. rhamnosus* ATCC 53103 in combination, no significant improvement was observed in the expression of genes involved in the antiviral immune response compared to that with *L. paracasei* LPC S01, *L. rhamnosus* ATCC 53103 or LF treatments alone (Figure 2). Notably, treatment of Caco-2 cells with *L. paracasei* DG together with LF significantly improved the activity of the antiviral immune response by increasing the expression of the *IFNA1, TLR3* and *IRF7* genes compared to that with *L. paracasei* DG treatment alone (Figure 2). The effectiveness of this combination was also significantly higher than that of LF alone in terms of upregulation of *IFNA1, IFNB1, TLR7, IFIH1, IRF7* and *MAVS* gene expression. These results suggest that *L. paracasei* DG ameliorates the *in vitro* immunostimulation activity of LF.

## *Inhibitory effect of the probiotic L. paracasei* DG *on SARS-CoV-2 replication in vitro.*

**Figure 3.**
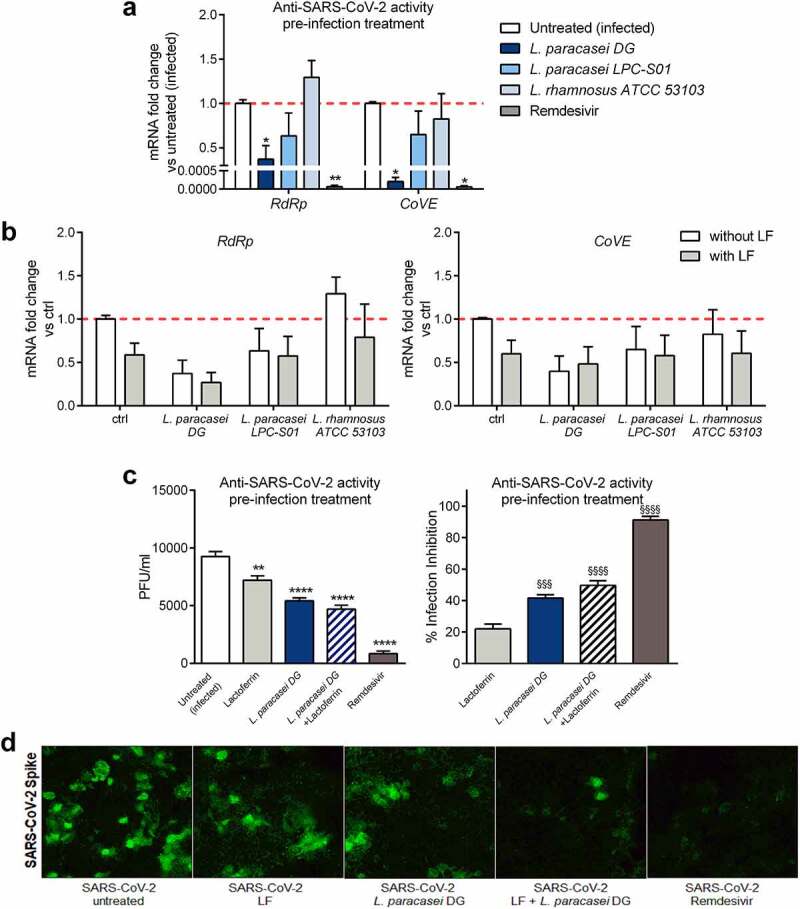
**The *L. paracasei* DG probiotic strain protects intestinal epithelial cells from SARS-CoV-2 infection *in vitro.*** Caco-2 cells were treated or not for 3 h with remdesivir as a positive control and (a) *Lacticaseibacillus* probiotic strains or (b) *Lacticaseibacillus* probiotic strains in combination or not with lactoferrin and then infected with SARS-CoV-2. SARS-CoV-2-specific gene expression was assessed by real-time qPCR 24 h post infection (n = 5). Data are shown as the relative fold change compared to the untreated (infected) control (arbitrarily set as 1) and presented as the mean + SD. *P < .05, **P < .01 vs untreated infected based on one-way ANOVA (followed by Dunnett’s multiple comparison test). (c) The SARS-CoV-2 titer was determined by plaque assay performed on harvested supernatants; the titer (plaque-forming units (PFU)/ml) in the supernatants as well as the percentage of inhibition of infection are represented. **P < .01, ****P < .0001 vs untreated infected; and ^§§§^*P* < .001, ^§§§§^*P* < .0001 vs LF based on one-way ANOVA (followed by Dunnett’s multiple comparison test). (d) Representative staining for SARS-CoV-2 spike in infected Caco-2 cells pre-treated or not with lactoferrin or *L. paracasei* DG alone or together with lactoferrin

To evaluate the antiviral activity of the different probiotic strains against SARS-CoV-2, an infection assay for SARS-CoV-2 was performed in Caco-2 cells. Prior to virus infection, cells were pre-treated with probiotic strains for 3 h and then infected with SARS-CoV-2 for 1 h (Supplementary Figure S1). Remdesivir treatment was used as a positive control. The expression level of the virus-specific genes encoding RNA-dependent RNA polymerase (*RdRp*) and the E gene (*CoVE*), critical for SARS-CoV-2 replication and assembly, was analyzed from total RNA obtained from harvested cells. As shown in Figure 3a, the expression of both genes was significantly reduced in *L. paracasei* DG-treated Caco-2 cells, indicating that pre-treatment with the probiotic strain could inhibit SARS-CoV-2 replication *in vitro*. Furthermore, when Caco-2 cells were pre-treated with LF and the three probiotic strains in combination, no significant improvement was observed in the inhibition of the expression of SARS-CoV-2 genes compared to that with LF pre-treatment alone (Figure 3b). Only pre-treatment of Caco-2 cells with *L. paracasei* DG together with LF tended to improve the antiviral activity of *L. paracasei* by further reducing the expression of the *RdRp* gene in comparison to that with LF alone pre-treatment. Thus, we next evaluated the SARS-CoV-2 titer in the harvested supernatants: pre-treatment with all tested conditions significantly diminished viral titers compared to that of the infection control, with *L. paracasei* DG alone or in combination with LF resulting in 41.5 ± 4.8% and 49.7 ± 4.4% inhibition of SARS-CoV-2 infection, respectively (Figure 3c). Moreover, immunofluorescence staining of Caco-2-infected cells for the viral spike glycoprotein confirmed that *L. paracasei* DG both alone and in combination with LF decreased SARS-CoV-2 infection and replication in Caco-2 cells (Figure 3d).

### L. paracasei DG pre-treatment protects against the inflammatory response triggered by SARS-CoV-2 in vitro

**Figure 4.**
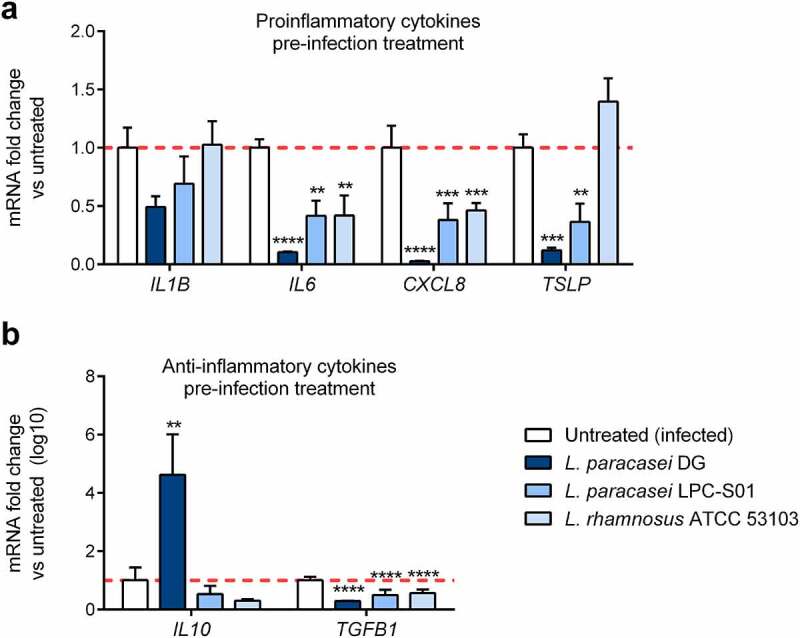
***Lacticaseibacillus* probiotic strains modulate cytokine production resulting from SARS-CoV-2 infection *in vitro***. Caco-2 cells were treated or not for 3 h with *Lacticaseibacillus* probiotic strains and then infected with SARS-CoV-2. The gene expression of (a) proinflammatory cytokines and (b) anti-inflammatory cytokines was assessed by real-time qPCR 24 h post infection (n = 5). Data are shown as the relative fold change compared to the untreated (infected) control (arbitrarily set as 1) and presented as the mean + SD. **P < .01, ***P < .001, ****P < .0001 vs untreated infected based on one-way ANOVA (followed by Dunnett’s multiple comparison test)

Proinflammatory cytokine levels are known to be elevated by SARS-CoV-2 infection, and in the most severe cases, patient prognosis can be markedly worsened along with the hyperproduction of proinflammatory cytokines. To determine whether pre-treatment with probiotic strains could be protective against the inflammatory response triggered by SARS-CoV-2 infection *in vitro*, the expression profile of inflammatory and anti-inflammatory cytokines in SARS-CoV-2-infected Caco2 cells pre-treated or not with *Lacticaseibacillus* cells was tested (Figure 4). The transcript levels of all the measured cytokines tended to be upregulated following infection with SARS-CoV-2 (data not shown). Notably, pre-treatment of infected Caco-2 cells with the *L. paracasei* DG strain significantly reduced the mRNA expression levels of the *IL6, CXCL8, TSLP* and *TGFB1* genes and augmented the transcript levels of *IL10* compared to those in the control (Figure 4a and 4b). This effect might be explained by the reduced viral load present following the antiviral activity of the *L. paracasei* DG strain; however, it could also be partially detected in infected Caco-2 cells pre-treated with the other probiotic strains, which showed minor antiviral activity. Overall, *L. paracasei* DG protective activity against the inflammatory response induced by SARS-CoV-2 was still effective but not improved when in combination with LF supplementation (Supplementary Figure S2).

## Discussion

Recently, a sizable number of reviews and opinion articles summarizing the antiviral effects of probiotics and their potential contribution in preventing and fighting virus infections with special focus on COVID-19 have been published. These publications support the administration of probiotics to patients with COVID-19 despite the absence of solid evidence supporting whether these treatments can prevent or treat this infectious disease. However, boosting the natural immunity of the population using probiotics before, during, or after COVID-19 infection is rational. In the present study, we report for the first time experimental evidence supporting the use of the *L. paracasei* DG strain for the prevention of SARS-CoV-2 infection.

Among the probiotic strains tested, *L. paracasei* DG was the most promising in terms of antiviral immunomodulatory activity and was able to induce the expression of *IFN* genes and genes involved in antiviral response signaling pathways, such as *TLR7, IFIH, IRF7* and *MAVS*. This finding is of special interest in the context of SARS-CoV-2 infection. Coronaviruses have various mechanisms to evade the innate immune response, especially by modifying the type I IFN response.^32^ In comparison with other respiratory viruses, SARS-CoV-2 induces a lower antiviral transcriptional response marked by low type I IFN levels and elevated chemokine expression.^33^ Furthermore, patients with severe COVID-19 exhibit an impaired type I IFN response and reduced viral clearance.^34^ In addition, whole-genome sequencing of SARS-CoV, MERS-CoV, and SARS-CoV-2 has shown that the SARS-CoV-2 genome contains more single-stranded RNA motifs that could interact with TLR7 than the SARS-CoV genome, indicating that TLR7 signaling might be even more relevant in the pathogenesis of COVID-19.^35^ In a case series of 4 young male patients with severe COVID-19, rare putative loss-of-function variants of X-chromosomal TLR7 were identified that were associated with impaired type I and II IFN responses.^36^

Our results showed not only the antiviral immune boosting activity of *L. paracasei* DG but also its ability to suppress SARS-CoV-2 replication *in vitro* by approximately 50%. Indeed, lactic acid bacteria (LAB) probiotics, such as *L. paracasei* DG, produce a wide variety of antimicrobial compounds, such as hydrogen peroxide, lactic acid and bacteriocin-like inhibitory substances, which have shown the ability to decrease viral loads.^37^ Proposed modes of antiviral action include direct interaction between the LAB and viruses, production of antiviral substances and stimulation of the host’s immune system.^17^ In the context of SARS-CoV-2 infection, lactobacilli may act as a barrier to viral penetration through several mechanisms.^38^ *Lactobacillus gasseri* Kx110A1 was reported to attenuate SARS-CoV-2 infection by inhibiting the expression of a disintegrin and metalloprotease 17 (ADAM 17),^39^ an enzyme that participates in ACE2 ectodomain shedding and that has been shown to play a role in the entry of SARS-CoV, since ADAM17 silencing was found to reduce SARS-CoV infection.^40^ Moreover, a computational study found that plantaricin, a bacteriocin secreted by *Lactiplantibacillus plantarum*, may possess SARS-CoV-2 antiviral activity by interacting with the receptor-binding domain (RBD) of the viral spike glycoprotein and thus blocking SARS-CoV-2 cellular entry.^41^ Some lactobacilli have been reported to release peptides with high affinity for ACE during milk fermentation.^42^ Although the mechanism supporting the antiviral activity of *L. paracasei* DG observed in this study is not known, we can speculate that the unique rhamnose-rich hetero-exopolysaccharide molecule that covers the cells of this bacterium may contribute to the peculiar cross-talk of DG with host cells.^43^ Indeed, a limitation of our study was the inability to determine the essential molecules produced by *L. paracasei* DG required to inhibit SARS-CoV-2 replication. Additionally, the detailed molecular mechanisms by which *L. paracasei* DG and LF inhibit viral replication need to be elucidated. The LF antiviral mechanisms vary among viruses, where it may bind either directly to the virus particle or to the host cell receptor or coreceptor.^44^ Based on our results, it seems that the *L. paracasei* DG and LF combination has additive antiviral and immunomodulatory effects, thus implying that they function independently without interfering with each other’s mechanism of action.

An unbalanced immune response, characterized by a weak production of type I interferons and an exacerbated release of proinflammatory cytokines, contributes to the severe forms of COVID-19.^45^ Moreover, low-grade chronic systemic inflammation accompanies several comorbidities that adversely affect the outcomes of patients with COVID-19.^46^ Our results show that prophylactic treatment with *L. paracasei* DG *in vitro* suppressed the inflammatory response triggered by SARS-CoV-2 infection in Caco-2 cells, as the transcript levels of the proinflammatory cytokines IL-6, CXCL8 and TSLP were reduced compared to those in the control. The anti-inflammatory potential of strain DG was also evidenced in previous *in vitro* experiments since it was demonstrated to significantly reduce the activation of NF-κB in Caco-2 cells,^47^ and its administration *in vivo* significantly diminished inflammatory cytokine levels and increased mucosal IL-10 levels in ulcerative colitis patients upon rectal administration.^48^ Thus, *L. paracasei* DG preventive use may contribute to the alleviation of the excessive inflammatory response induced by SARS-CoV-2 infection.

Previous studies using several probiotic species and strains showed that their immunomodulatory effects were strain specific.^49^ Our results revealed different antiviral immunity activities as well as distinct levels of inhibition of SARS-CoV-2 among the three tested probiotic strains. In particular, we found that the antiviral properties of *L. paracasei* DG were better than those of LPC-S01, a probiotic strain belonging to the same species as strain DG, confirming that the immunological effects of probiotics are strain-specific features.^12^ In addition, *L. paracasei* DG displayed enhanced activities compared to those of *L. rhamnosus* GG, the most extensively studied and one of the most widely used probiotics worldwide, which has been documented to exert immunomodulatory properties.^50^ Since the ability to affect host immune responses is primarily strain specific, the choice of effective strains of probiotics is fundamental for the development of novel and targeted approaches for gut microbiota modulation as a preventive strategy against COVID-19. Here, we propose *L. paracasei* strain DG as a promising candidate for this purpose.

The efficacy of probiotics in COVID-19 patients remains to be proven, and the issue is under debate.^51,52^ Several clinical studies of probiotic intervention in COVID-19 are underway.^53^

In conclusion, our work showed that the probiotic strain *L. paracasei* DG is a promising candidate that exhibits prophylactic potential against SARS-CoV-2 infection. The characterization of *L. paracasei* DG mechanisms that enable inhibition of SARS-CoV-2 replication, as well as of its effect *in vivo*, represent an important future research direction.

## Supplementary Material

Supplemental Material

## Data Availability

The data presented in this study are available on request from the corresponding author.
