# Supplementary material for: Lacticaseibacillus paracasei DG enhances the lactoferrin anti-SARS-CoV-2 response in Caco-2 cells
Source: Gut Microbes. 2021 Aug 7;13(1):1961970. doi: 10.1080/19490976.2021.1961970 (PMC8354669; doi:10.1080/19490976.2021.1961970)
Supplement: Supplemental Material [file KGMI_A_1961970_SM5898.zip › Supplementary Materials and figures rev2 engl edit.docx]

**Supplementary Materials and Methods**

***Viral Stock Preparation and Titration***

The viral titre was determined by the plaque assay method. Briefly, confluent VERO E6 cells in 24-well plates (Costar, Merck, Italy) were inoculated with serial 10-fold dilutions of the virus stock for 1 h. Thereafter, the virus inoculum was removed, and the cells were washed with PBS (Gibco-Thermo Fisher Scientific, Waltham, USA) and then incubated with fresh medium containing 0.6% carboxymethylcellulose (CMC, Merck). The cells were fixed 72 h post infection with 5% w/v formaldehyde (Merck) and stained with crystal violet (Merck). The viral titre was measured as plaque-forming units (PFU/mL) based on the plaques formed in cell culture upon infection.

***RNA Extraction and Real-Time RT-PCR***

Total RNA was isolated using an E.Z.N.A.® Total RNA Kit I (Omega Bio-Tek, tebu-bio, Italy) following the manufacturer's instructions. Contaminating DNA was removed by incubation with a RNase-free DNase I kit (Omega Bio-tek). Complementary DNA synthesis and amplification were performed using an iTaq^TM^ Universal Probes One-Step Kit (Bio-Rad, Milan, Italy) according to the manufacturer’s directions in an ABI PRISM 7000 Sequence Detection System (Applied Biosystems). The expression of the target gene was normalized to the expression of the GAPDH reference gene. The specific forward and reverse primers used are summarized in Supplementary Table S1. Data are presented as the mean fold change over the control.

***Immunofluorescence***

Twenty-four hours post infection, cells were fixed with 4% paraformaldehyde and blocked with 2.5% BSA in 1x TBS buffer containing 0.5% Triton for 1 h at room temperature. The cells were washed and incubated with a primary antibody (anti-SARS-CoV-2 Spike antibody, GeneTex GTX135360) for 1 h at RT, followed by incubation with a secondary antibody (Alexa Fluor™ 488 goat anti-rabbit IgG, Life Technologies) for 1 h at room temperature. Viral protein expression was visualized using confocal microscopy (Nikon A1).

**Supplementary Table S1. Primers used in the study.**

| **Gene** | **5'-->3' forward sequence** | **5'-->3' reverse sequence** | **T_a_ °C** |
| --- | --- | --- | --- |
| *GAPDH* | GACACCCACTCCTCCACCTTT | TTGCTGTAGCCAAATTCGTTGT | 60 |
| *IFNA1* | TTCAGGGGCATCAGTCCCTA | CCGTCCATTCCTTGATTTGGTT | 60 |
| *IFNB1* | TCTCCTGTTGTGCTTCTCCAC | GCCTCCCATTCAATTGCCAC | 60 |
| *IL1B* | CTGAGCTCGCCAGTGAAATG | TGTCCATGGCCACAACAACT | 60 |
| *IL6* | GTCCAGTTGCCTTCTCCCTGG | CCCATGCTACATTTGCCGAAG | 60 |
| *IL10* | GTGAAAACAAGAGCAAGGCCG | TAGAGTCGCCACCCTGATGT | 60 |
| *TGFB1* | ACTGCGGATCTCTGTGTCAT | AGAGTCCCTGCATCTCAGAGT | 60 |
| *CXCL8* | TGGACCCCAAGGAAAACTGG | ATTTGCTTGAAGTTTCACTGGCA | 60 |
| *TSLP* | AAGGCAACAGCATGGGTGAA | GGGAACATACGTGGACACCC | 60 |
| *TLR3* | CCTTTTGCCCTTTGGGATGC | TGAAGTTGGCGGCTGGTAAT | 60 |
| *TLR7* | CCTTGTGCGCCGTGTAAAAA | GGGCACATGCTGAAGAGAGT | 60 |
| *MAVS* | GCAATGCCGTTTGCTGAAGA | CGCCGCTGAAGGGTATTGAA | 60 |
| *IFIH1* | GCATATGCGCTTTCCCAGTG | CTCTCATCAGCTCTGGCTCG | 60 |
| *IRF3* | GAGCTGTGCTGGCGAGAAG | CTCTCCAGGAGCCTTGGTTG | 60 |
| *IRF7* | CCATCGGCTTTTGGGTCTGT | TTCCCATGGTCCGGCCTC | 60 |
| *CoVE* | ACAGGTACGTTAATAGTTAATAGCGT | ATATTGCAGCAGTACGCACACA | 60 |
| *RdRp* | ATGAGCTTAGTCCTGTTG | CTCCCTTTGTTGTGTTGT | 60 |

**Supplementary Figures**

**
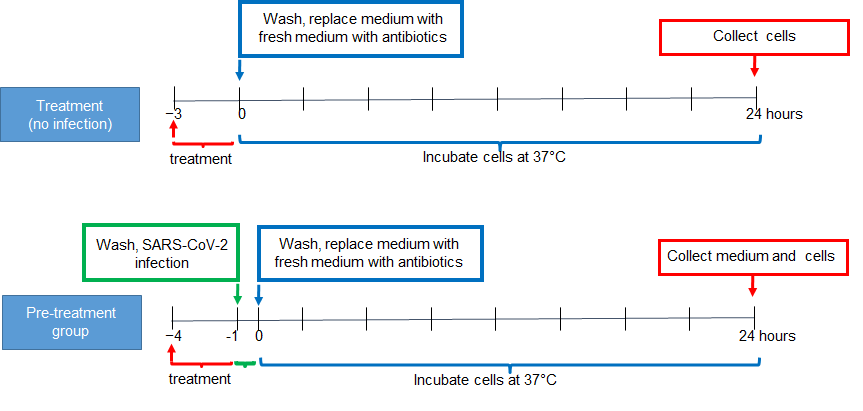
**

**Supplementary Figure S1.** Experimental design: treatments (probiotics, lactoferrin or their combination) and SARS-CoV-2 infection of the Caco2 cell line.

**Supplementary Figure S2. Effect of the lactoferrin and probiotic combination on cytokine production resulting from SARS-CoV-2 infection *in vitro***. Caco-2 cells were treated or not for 3 h with or without lactoferrin in combination with *Lacticaseibacillus* probiotic strains and then infected with SARS-CoV-2. The gene expression of (a) proinflammatory cytokines and (b) anti-inflammatory cytokines was assessed by real-time qPCR 24 h post infection (n=5). Data are shown as the relative fold change compared to the untreated (infected) control (arbitrarily set as 1) and presented as the mean ± SD. ****P*<0.001 vs *L. paracasei DG* without LF; and ^§§^*P*<0.01, ^§§§^*P*<0.001 vs LF based on one-way ANOVA (followed by Bonferroni’s multiple comparison test).
